# Neural dynamics of social verb processing: an MEG study

**DOI:** 10.1093/scan/nsae066

**Published:** 2024-12-27

**Authors:** Lucia Amoruso, Sebastian Moguilner, Eduardo M Castillo, Tara Kleineschay, Shuang Geng, Agustín Ibáñez, Adolfo M García

**Affiliations:** Cognitive Neuroscience Center (CNC), University of San Andres, Buenos Aires C1011ACC, Argentina; Basque Center on Cognition, Brain and Language (BCBL), San Sebastian 20009, Spain; Ikerbasque, Basque Foundation for Science, Bilbao 48009, Spain; Cognitive Neuroscience Center (CNC), University of San Andres, Buenos Aires C1011ACC, Argentina; Global Brain Health Institute (GBHI), University of California San Francisco (CA94158), United States; Trinity College Dublin (TCD), Dublin D02DP21, Ireland; Magnetoencephalography Laboratory, Advent Health for Children, Orlando, FL 32804, United States; Magnetoencephalography Laboratory, Advent Health for Children, Orlando, FL 32804, United States; Brain, Language and Computation Lab, The Hong Kong Polytechnic University, Kowloon, Hong Kong, China; Cognitive Neuroscience Center (CNC), University of San Andres, Buenos Aires C1011ACC, Argentina; Global Brain Health Institute (GBHI), University of California San Francisco (CA94158), United States; Trinity College Dublin (TCD), Dublin D02DP21, Ireland; Latin American Brain Health Institute (BrainLat), Universidad Adolfo Ibáñez, Santiago 8320000, Chile; Cognitive Neuroscience Center (CNC), University of San Andres, Buenos Aires C1011ACC, Argentina; Global Brain Health Institute (GBHI), University of California San Francisco (CA94158), United States; Trinity College Dublin (TCD), Dublin D02DP21, Ireland; Departamento de Lingüística y Literatura, Facultad de Humanidades, Universidad de Santiago de Chile, Santiago 9170022, Chile

**Keywords:** social concepts, verbs, magnetoencephalography, oscillations, temporal decoding

## Abstract

Human vocabularies include specific words to communicate interpersonal behaviors, a core linguistic function mainly afforded by social verbs (SVs). This skill has been proposed to engage dedicated systems subserving social knowledge. Yet, neurocognitive evidence is scarce, and no study has examined spectro-temporal and spatial signatures of SV access. Here, we combined magnetoencephalography and time-resolved decoding methods to characterize the neural dynamics underpinning SVs, relative to nonsocial verbs (nSVs), via a lexical decision task. Time-frequency analysis revealed stronger beta (20 Hz) power decreases for SVs in right fronto-temporal sensors at early stages. Time-resolved decoding showed that beta oscillations significantly discriminated SVs and nSVs between 180 and 230 ms. Sources of this effect were traced to the right anterior superior temporal gyrus (a key hub underpinning social conceptual knowledge) as well as parietal, pre/motor and prefrontal cortices supporting nonverbal social cognition. Finally, representational similarity analyses showed that the observed fronto-temporal neural patterns were specifically predicted by verbs’ socialness, as opposed to other psycholinguistic dimensions such as sensorimotor content, emotional valence, arousal, and concreteness. Overall, verbal conveyance of socialness seems to involve distinct neurolinguistic patterns, partly shared by more general sociocognitive and lexicosemantic processes.

## Introduction

Language is a species-specific facilitator of social interaction (Pexman et al. 2023). Accordingly, the human brain seems to possess specialized mechanisms to process socially laden words, crucially including social verbs (SVs). While spectro-temporal neural measures have revealed core mechanisms underpinning other lexical categories such as action verbs (Niccolai et al. 2016, Klepp et al. 2019) and object nouns (Amoruso et al. 2021), no study has targeted SVs, limiting neurocognitive accounts of sociolinguistic skills. Facing this gap, we combined magnetoencephalography (MEG) and decoding methods to reveal key neural signatures of SVs relative to nonsocial verbs (nSVs).

Human vocabularies encompass multiple units that evoke interpersonal behaviors, traits, or events (Diveica et al. 2023). Core social concepts are expressed through SVs, which signal dyadic or gregarious conducts, such as communicative (e.g. *promise*), prosocial (e.g. *cooperate*), and antisocial (e.g. *harm*) actions. Compared to nonsocial events, social interactions elicit stronger beta (15–25 Hz) power decreases (Kourtis et al. 2010, Menoret et al. 2014) and greater theta (4–8 Hz) activity—an effect that is observed in infancy and becomes more pronounced with age (van der Velde et al. 2021). As regards their timing, socialness effects typically involve early modulations (< 350 ms) likely reflecting social salience detection or social categorization (Proverbio et al. 2008, Schacht and Vrticka 2018, Lee Masson and Isik 2023, Moreau et al. 2023). Yet, though informative, this evidence fails to reveal distinct neurophysiological correlates of *social words* in general, and SVs, in particular. Given the centrality of words in interpersonal dynamics, this undermines the formulation of comprehensive models of semantic memory (Ralph et al. 2017, Binney and Ramsey 2020) and social cognition at large (Adolphs 2010, Frith and Frith 2010).

At the anatomical level, social concepts recruit dedicated systems including the superior anterior temporal lobe (ATL), particularly in the right hemisphere (Zahn et al. 2007, Olson et al. 2013, Binney et al. 2016). This has been replicated by the few neuroimaging studies targeting SVs (Spunt et al. 2010, Lin et al. 2015), showing additional involvement of prefrontal and parieto-temporal regions subserving nonverbal socio-cognitive processes, such as action comprehension and theory of mind (ToM). Thus, socialness appears to represent a distinct dimension of conceptual knowledge, engaging neural circuits that underpin interpersonal abilities at large (Lopes da Cunha et al. 2023). The right ATL has been further shown to participate in word retrieval via early beta power decreases (Abel et al. 2016, Cao et al. 2022). Since similar temporal effects have been reported during social salience detection and/or social categorization (Ibanez et al. 2012), early beta modulations in the ATL might mediate the integration of lexico-semantic retrieval and social information processing. Yet, this hypothesis, in general, and its potential extension to SVs, in particular, awaits empirical testing.

Robust testing of this conjecture requires both item-level and trial-by-trial classification approaches. Most neurolinguistic studies focus on neural activity changes averaged across trials, using mean responses to draw conclusions about all words within a certain category. This approach can introduce bias, mask, or even eliminate early short-lived effects due to the variability in the psycholinguistic features of individual words within categories (Shtyrov and Stroganova 2015). Furthermore, theories of social semantic knowledge diverge regarding the contribution of other dimensions (e.g. sensorimotor content, emotional valence, arousal, and concreteness) that could covary with socialness and explain preferential activations during lexico-semantic processing (Pexman et al. 2023). Promisingly, recent neural decoding methods, such as representational similarity analysis (RSA) and supervised machine learning, offer complementary insights to address these limitations (Haxby et al. 2014, Popal et al. 2019). RSA allows examining the extent to which words’ psycholinguistic features, including socialness, are encoded in observed neural patterns at the item-level by comparing similarity structures of neural responses to conceptual models (Tamir et al. 2016). In contrast, supervised machine learning classifiers allow categorizing neural data into predefined categories (e.g. SVs and nSVs) on a trial-by-trial basis, revealing how well and when different stimulus types can be read-out from brain signals (Hastie et al. 2017). A timely opportunity thus arises to investigate the consistency of social information processing across SVs.

In summary, current approaches to social semantics face major gaps in modeling spectro-temporal and spatial dimensions of SV processing, especially at the single-trial level. This MEG study tackles such gaps. We asked healthy participants to perform lexical decisions on SVs and nSVs (relative to pseudo-verbs) and tracked underlying oscillatory dynamics and their neural sources. Using time-resolved decoding methods, we further evaluated: (i) the periods of maximal discriminability of SVs and nSVs using supervised machine learning, and (ii) whether word-differential neural patterns were uniquely related to socialness vis-à-vis other semantic models (e.g. sensorimotor content, emotional valence, arousal, and concreteness) using RSA. Building on previous evidence, we raised three hypotheses. First, we predicted that, relative to nSVs, SVs would modulate beta (and possibly theta) oscillations at early stages (<300 ms). Second, we hypothesized that such oscillatory effects would be traceable to the activity of the ATL as a key social semantic hub and potentially engage other brain regions involved in social cognition. Finally, we anticipated above-chance decoding of SVs and nSVs from relevant neural patterns. With this novel approach, we seek to understand how the human brain accesses social information conveyed through language.

## Materials and methods

### Participants

Sixteen English-speaking volunteers participated in the study. However, two participants were removed from the final analyses due to major artifacts in the recordings, yielding a final sample of 14 participants (8 women) with a mean age of 23.57 (*SD* = 7.33, range = 18–44 years old) and an average of 15.78 (*SD* = 2.45) years of education. All participants were right-handed, as assessed via the Edinburgh Handedness Inventory (Oldfield 1971), and had normal or corrected-to-normal vision. They were healthy and had no family history of neurological or psychiatric disease. Prior to the study, participants provided written informed consent. The study protocol was approved by the Institutional Review Board for the Protection of Human Research Participants at Florida Hospital (Orlando, USA) and was carried out following the Code of Ethics of the World Medical Association (Declaration of Helsinki) for experiments involving humans.

### Experimental design

All participants performed a lexical decision task comprising 160 trials. The first 10 integrated a practice block (for task familiarization) and were omitted from the actual experiment. Of the 150 remaining trials, 100 ended with real English verbs, whereas the other 50 ended with pseudoverbs. Although the latter were excluded from analyses, they served to confirm task compliance and to guarantee attention by requiring linguistic decisions on a trial-by-trial basis. To create the pseudoverbs, 50 real words were selected from the list of real verbs and a single letter was replaced to form a phonotactically and graphotactically valid non-English word (Cervetto et al. 2021).

Of the 100 trials containing real verbs, half of them featured SVs, rich in interpersonal associations (e.g. *thank, educate*, and *forgive*); while the other half featured nSVs, with low to null interpersonal associations (e.g. *type, stretch*, and *unpack*). Crucially, as isolated English words can manifest different grammatical categories (e.g. *help* can be either a noun or a verb), target items were shown with a grammatical context (i.e. *I am*) that forced their interpretation as verbs (e.g. *I am helping*). All items, including verbs and pseudoverbs, were presented in their inflected forms.

Verbs’ socialness was determined through the largest database of social ratings, totaling 8388 English words, including 1343 verbs (Diveica et al. 2023). These ratings were obtained by asking participants to quantify the social relevance of word meanings on a Likert scale ranging from 1 (minimally social) to 7 (maximally social). For verbs, the focus was on the extent to which they feature social behaviors or interactions. Based on these ratings, we classified verbs as social if rated ≥∼4 and as nonsocial if rated ≤∼3. Socialness was significantly higher [*t*(98) = 21.5, *P* < .0001; *d* = 4.3] for SVs (*M *= 5.05; *SD* = 0.68) than for nSVs (*M *= 2.64; *SD* = 0.4). Both sets were controlled for orthographic length, phonemic length, frequency, familiarity, orthographic neighbors, age of acquisition, valence, arousal, and concreteness—based on Davis (2005), Kuperman et al. (2012), Warriner et al. (2013), Brysbaert et al. (2014), as well as perceptual and motor properties—based on norms from the Lancaster database (Lynott et al. 2020). Specifically, the perceptual content was calculated as the mean ratings from the auditory, gustatory, olfactory, visual and haptic subscales; while the motor content was computed as the mean of the foot, hand, head, mouth, and torso subscales. See Table 1 for detailed descriptive statistics.

**Table 1.** Lexical features of the social and nonsocial verbs.

Lexical variables	Social verbs	Non-social verbs	*t*-value	*P*-value	Cohen’s *d*
Orthographic length ^a^	8.88 (1.13)	8.62 (1)	1.21	.22	0.24
Phonemic length ^a^	7.04 (1.37)	6.76 (1.2)	1.01	.31	0.22
Frequency ^a^^,^ ^b^	24.29 (27.88)	16.65 (25.54)	1.43	.15	0.28
Familiarity ^a^^,^ ^c^	518.3 (42.97)	503.1 (56.99)	1.1	.27	0.3
Orthographic neighbors ^a^	1.44 (2.43)	2.26 (3.36)	−1.39	.16	−0.27
Age of acquisition ^d^	7.61 (2.03)	8.36 (2.3)	−1.73	.08	−0.35
Concreteness ^e^	2.77 (0.7)	3.04 (0.65)	−1.93	.055	−0.38
Motor content ^f^	1.63 (0.49)	1.54 (0.43)	0.91	.36	0.18
Perceptual content ^f^	1.29 (0.32)	1.37 (0.35)	−1.28	.2	−0.25
Emotional valence ^g^	5.46 (1.61)	5.07 (0.94)	1.49	.13	0.3
Arousal ^g^	4.73 (0.91)	4.21 (0.83)	3	.003	0.6

Data presented as mean (SD). Comparisons between social and nonsocial verbs were made via two-tailed independent samples *t*-tests.

a Data obtained through N-Watch (Davis 2005).

b Data averaged from the CELEX (total), the British National Corpus Word Frequency, and the Sydney Morning Herald Word Frequency databases.

c Data from the MRC database.

d Data from Kuperman et al. (2012).

e Data from Brysbaert et al. (2014).

f Data from Lynott et al. (2020).

g Data from Warriner et al. (2013).

**Figure 1.**
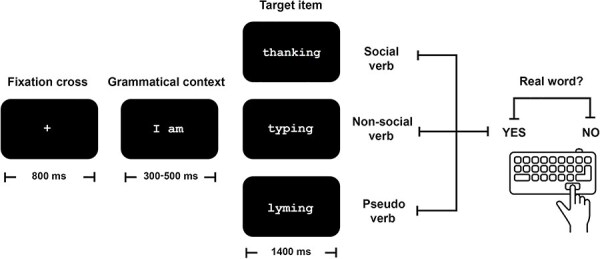
Lexical decision task. Participants viewed a fixation cross, followed by the words “*I am*” and then the target item. The latter could be a social verb, a nonsocial verb, or a pseudoverb. In each trial, participants had to choose if the target item was a real word or not.

Words were displayed in white lowercase Courier font (size 48) against a black background using E-Prime 3.0 software (Psychology Software Tools, Pittsburgh, PA, USA). The display was situated at a distance of 1.5 m from participants, and each stimulus subtended a maximum visual angle of 4º. Each trial began with an 800 ms fixation cross at the center of the screen, followed by the grammatical context *I am* displayed for a random period between 300 and 500 ms. Grammatical context duration was balanced across SV and nSV conditions. Immediately after context presentation, the sentence-final target item (verb or pseudoverb) was presented for up to 1400 ms in its inflected form (Fig. 1). The target disappeared upon the participant’s button press, which triggered the next trial. Trials were presented in a pseudorandomized order, ensuring that no more than three target stimuli from the same category were presented consecutively. The entire task lasted for ∼25 min.

### MEG data acquisition

MEG recordings were acquired at the Magnetoencephalography Laboratory in Florida Hospital for Children, using a 306-channel Elekta Neuromag TRIUX system placed in a magnetically shielded room with three layers (Vacuumschmelze GmbH & Co, Germany). The research followed recommended practices for MEG data acquisition (Gross et al. 2013). The MEG system included 204 orthogonal planar gradiometers and 102 magnetometers situated over 102 locations and housed in a helmet-shaped device. Participants were in a supine position during the recordings, and their heads were covered by the MEG sensor array. Participants’ head position inside the helmet was continuously monitored using five head-position indicator coils. The standard fiducial landmarks (i.e. left and right pre-auricular points and nasion) plus ∼300 additional points registered over the scalp and eyes/nose contours were digitalized using a 3D digitizer (Polhemus, VT, USA). During recordings, a closed-loop real-time noise cancellation system (‘MaxShield’, Elekta Neuromag, Helsinki, Finland) was used, and data were collected at a sampling rate of 1 kHz and online filtered at a bandwidth of 0.1–330 Hz.

### MEG data preprocessing

Continuous MEG data preprocessing, external magnetic noise elimination, and head movement correction were performed via the spatio-temporal signal space separation (tSSS) method (Taulu and Simola 2006) implemented offline on Maxfilter 2.2 (Elekta-Neuromag). Further analyses were run on MatlabR_2014B (The MathWorks, Inc., Natick, MA, USA) and FieldTrip Toolbox [version 20 170 911] (Oostenveld et al. 2011). Recordings were downsampled to 500 Hz and segmented from 1000 ms before to 1500 ms after target word onset. The extended period preceding the onset of the target word was selected to capture a clear baseline interval during which no linguistic stimuli were presented—namely, during the presentation of the fixation cross (−750 to −450). A semi-automated method was used to eliminate segments containing electromyographic artifacts, SQUID jumps, and flat signals. Then, heartbeat and electro-oculogram (EOG) artifacts were identified and linearly removed from the recordings via the FastICA method (Hyvarinen 1999, Jung et al. 2000). The number of heartbeat and EOG components removed across participants varied from 1 to 3 and from 1 to 2, respectively. After data cleaning, which involved rejecting trials with incorrect responses, the number of remaining trials did not significantly differ between conditions [SVs: *M* = 43.85, *SD* = 2.17; nSVs: *M* = 43.07, *SD* = 2.49; *t*(13) = 1.22, *P* = .24, *d* = 0.32].

### Statistical analysis

#### Sensor-level analysis

Oscillatory dynamics were examined via time-frequency representation (TFR) analysis. The TFRs were calculated on the clean MEG data using a fixed 500 ms sliding Hanning window in time steps of 10 ms and frequency steps of 1 Hz, for a time window of −1000 ms to 1500 ms and a frequency range of 1–30 Hz. Power was separately estimated for each orthogonal direction of a gradiometer pair and further combined for a total of 102 measurement sensors. Power was calculated as the change relative to a ∼300 ms prestimulus baseline extending throughout the presentation of the fixation cross (−750 to −450). Statistical differences between conditions were evaluated using cluster-based permutation tests (Maris and Oostenveld 2007). The permutation *P*-value was obtained with the Monte-Carlo method (1000 permutations). The alpha threshold for significance testing was set at a *P*-value <5% (two-tailed).

While previous neurophysiological studies have not specifically examined the temporal dynamics involved in processing SVs and nSVs using linguistic stimuli, there is evidence from M/EEG studies that have manipulated the socialness of nonverbal stimuli. These studies (Proverbio et al. 2008, Schacht and Vrticka 2018, Lee Masson and Isik 2023, Moreau et al. 2023), primarily highlight social effects during early stages (<350 ms). Consequently, we focused our analysis not only on an early time-window (∼150–350 ms) but also conducted an exploratory analysis on a later period (∼350–650 ms) due to the lack of linguistic evidence on the domain. Time points within these windows were averaged to increase statistical power. To investigate the spectral dynamics potentially underlying socialness effects, we separately examined integer frequency bins at 6 Hz (theta), 10 Hz (alpha), and 20 Hz (beta), giving their critical role in supporting neural communication within sociocognitive networks (Kourtis et al. 2010, Moreno et al. 2013, Menoret et al. 2014, van der Velde et al. 2021).

#### Source reconstruction of sensor-level effects

Significant effects identified at the sensor level were subjected to source reconstruction. To this end, we used a fixed anatomical template for every subject (MNI152 NLIN 2009). The forward model was calculated using the Boundary Element Method implemented in the MNE suite (Gramfort et al. 2014) for three orthogonal tangential current dipoles, placed on a 5 mm grid covering the whole brain. The forward model was then reduced to the two primary components of the highest singular value for each source. Both planar gradiometers and magnetometers were used for source inverse modeling, normalizing each sensor’s signal by its noise standard deviation estimated from the baseline period. Brain source activity was calculated with a Linearly Constrained Minimum Variance beamformer approach (Van Veen et al. 1997), which deals well with location bias (Sekihara et al. 2005). To derive beamformer weights, a common filter was computed by combining the cross-spectral density matrices from the time-frequency window that showed significant sensor-level effects and an equally sized baseline period prior to target word onset (i.e. presentation of the fixation cross). Brain maps were transformed to the standard Montreal Neurological Institute (MNI) template using the spatial normalization algorithm integrated into Statistical Parametric Mapping (SPM8) (Ashburner and Friston 1999), leading to subject-level normalized power maps in the MNI space.

The location-comparison method (Bourguignon et al. 2018) was used to compare SVs and nSVs conditions. This method involves generating bootstrap group-averaged maps to establish a distribution of location differences between local maxima in the two conditions, while testing the null hypothesis that the distance is zero. MNI coordinates of local maxima (i.e. sets of contiguous voxels exhibiting higher power than neighboring voxels within each map) were obtained for each condition. Random sampling with replacement was used to select pairs of maps from the available subjects and create group-averaged maps. Within each group-averaged map, the local maximum closest to the mean coordinates of genuine parametric maps’ maxima was identified and coordinate differences were recorded. This process was iterated 1000 times to construct a sample distribution of local maximum coordinates from the group-averaged parametric maps and determine a threshold for statistical significance (*P* < .05) based on the 95th percentile of the permutation distribution. Any supra-threshold MEG peaks were interpreted as indicative of brain regions contributing to sensor-level effects. The contribution of each identified source was further evaluated by comparing MEG peaks between SVs and nSVs conditions using paired-sample *t*-tests.

#### Sensor-level temporal decoding

A time-resolved decoding approach was employed to determine in a fine-grained fashion when and for how long differences in SV and nSV categories achieved maximal discrimination. For each subject, a linear discriminant analysis (LDA) classifier (Hastie et al. 2017) was trained on each trials’ time point (−100 ms to 500 ms) using the category-sensitive gradiometers captured by the sensor-level analysis. To obtain a reliable estimate of the model’s performance and minimize potential overfitting, we used a 5-fold cross-validation approach. This method ensures that the model generalizes well to unseen data by dividing the dataset into five subsets, training the model on four subsets, and validating it on the remaining one (Nara et al. 2023). Classifier performance was evaluated using the area under the curve (AUC), which was averaged across folds for each time point and participant. Statistical significance was assessed by computing an empirical *P*-value for each time point using a permutation test (1000 permutations), in which SVs and nSVs labels were randomly shuffled to create a null distribution of AUC values. Multiple comparisons were controlled using false discovery rate (FDR) corrections (Hochberg and Benjamini 1990). All analyses were implemented in the Python library Scikit-learn (Pedregosa et al. 2011).

#### Representational similarity analysis

RSA (Kriegeskorte et al. 2008) was used to test whether neural patterns identified by the cluster-based analysis could be uniquely predicted by words’ social content or by other psycholinguistic features. First, candidate conceptual models were created to evaluate the prediction of socialness and other dimensions of interest. Models were computed to reflect the pairwise Euclidean distances in verbs based on: (i) socialness ratings from the Diveica et al. (2023); (ii) perceptual and (iii) motor content, derived from the Lancaster database (Lynott et al. 2020); (iv) arousal and (v) emotional valence, based on ratings from Warriner et al. (2013); (vi) concreteness, as extracted from Brysbaert et al. (2014); (vii) frequency, obtained from averaging the CELEX (total), the British National Corpus Word Frequency, and the Sydney Morning Herald Word Frequency databases; and (viii) AoA rating derived from Kuperman et al. (2012). Potential dependencies among the socialness model and the other predictor matrices were evaluated prior to RSA. The socialness dissimilarity matrix was not correlated with the perceptual (*r* = −0.01), motor (*r* = 0.06), concreteness (*r* = 0.09), valence (*r *= 0.13), arousal (*r* = 0.04), frequency (*r* = 0.04), or AoA (*r* = −0.01) models, indicating that they captured different psycholinguistic aspects of the words. Second, for each subject and time point (−100 ms to 500 ms), the correlation distance (1 minus Pearson correlation) was computed between MEG activity across trial pairs. This process generated subject-level, time-resolved neural representational dissimilarity matrices (RDMs), capturing the pairwise dissimilarities among the brain representations of words over time. Beta (20 Hz) power values from individual trials at significant gradiometers were used at input to compute the neural RDMs. Third, each subject’s neural RDM at each time point was correlated with the different conceptual RDMs using partial Spearman correlations, while partialling out all other RDM models. Finally, all correlation values were Fisher-transformed and then subjected to one-sided Wilcoxon signed-rank test against zero to identify significant correlations across time (Anderson et al. 2019, Kaiser and Nyga 2020). FDR correction for multiple comparisons was applied across time points and models. RSA analysis was performed with custom scrips using MNE-Python.

#### Code accessibility

All the data that support the findings of this study as well as the code for data preprocessing and analysis are available online at https://osf.io/j6r2t/.

## Results

### Behavioral results

Mean accurate response rates in the lexical decision task were 93% for SVs (*SD* = 0.038) and 95% (*SD* = 0.027) for nSVs. A Shapiro–Wilk test showed that accuracy scores for both SVs (0.93, *P* = .32) and nSVs (0.97, *P* = .9) had normal distributions. A paired sample *t*-test revealed that accuracy did not differ between conditions [*t*(13) =  −1.44, *p* = 0.17, *d* = −0.38].

Mean response times were 683.78 ms (*SD* = 39.66 ms) for SVs and 676.31 ms (*SD* = 41.55 ms) for nSVs. A Shapiro–Wilk test showed that response times were not normally distributed for either SVs (0.69, *P* < .05) or nSVs (0.71, *P* < .05). Thus, we performed a Wilcoxon signed-rank nonparametric test that revealed no significant differences between conditions (*W* = 78, *P* = .11, RBC = 0.48).

### Time-frequency results

**Figure 2.**
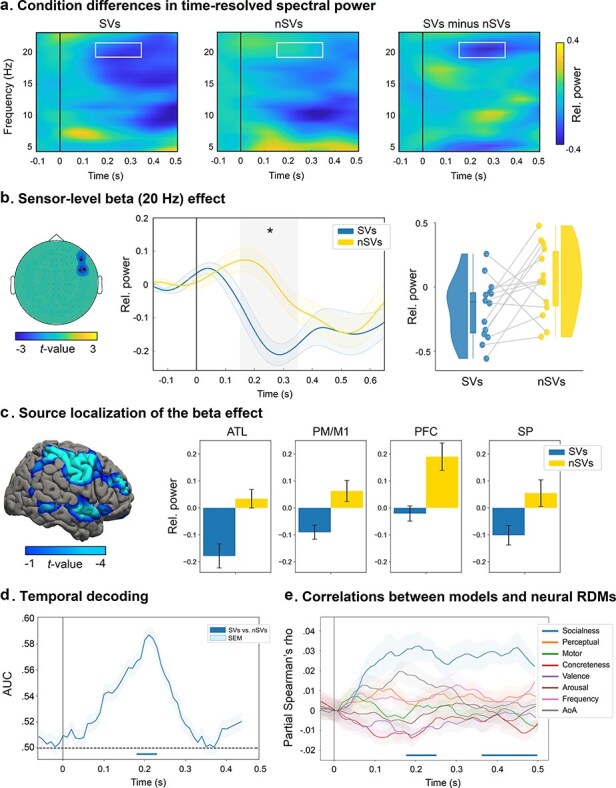
Spatiotemporal signatures of social verb processing. (a) Time-frequency representations for SVs and nSVs conditions, as well as their difference (SVs minus nSVs), averaged across significant sensors identified through cluster-based analysis in the beta (20 Hz) band. White rectangles indicate the cluster’s temporal window. (b) Topography and sources of the effect. The left inset shows the topography of the negative cluster involving fronto-temporal sensors in the right hemisphere (“MEG1322 + 1323,” “MEG1412 + 1413,” and “MEG1442 + 1443”). The middle inset displays beta power dynamics (relative to baseline) within the cluster for each condition separately. The right inset shows raincloud plots depicting subject-level effects, with individual points representing mean beta power within significant sensors in the nSVs and SVs conditions. (c) Source reconstruction of the beta cluster. Localization of the beta effect spanned the right superior ATL, motor/premotor, prefrontal and superior parietal cortices. (d) Decoding performance as a function of time. Classification AUC and SEM across subjects. Dotted black line at AUC 0.5 indicates chance level. Significant period of maximal decoding is highlighted below, occurring between 180 and 230 ms (*P* < .05, FDR-corrected). (e) Partial Spearman correlations between RDM predictive models and neural RDMs at every time point. Shaded regions represent SEM across subjects. Significant time points are highlighted below, occurring between 180–250 ms and 360–500 ms (*P* < .05, FDR-corrected).

A significant difference (Monte Carlo *P* = .018, two-tailed) was found in the beta band (20 Hz) during the early time window (150–350 ms), with stronger power decreases for SVs than nSVs (Fig. 2a). This difference was underscored by a negative cluster spanning right fronto-temporal combined gradiometers (Fig. 2b). No other clusters were found in any of the other frequency bands [i.e. theta (6 Hz) and alpha (10 Hz)] or time windows (i.e. early and late). As depicted in the raincloud plot (Fig. 2b), this effect was observed consistently across most participants, with 11 of them (78.5%) exhibiting the same direction of modulation.

### Source reconstruction of sensor-level results

Significant oscillatory effects at the sensor level were reconstructed considering the frequency-band and time-window highlighted by the significant cluster. The early beta (20 Hz) effect was source-localized in the right hemisphere, with the strongest socialness effect (SVs versus nSVs) peaking in the superior ATL (*t* = −3.85, *P* = .003, *d* = 1.1), followed by prefrontal (*t* = −3.01, *P* = .01, *d* = 0.87), ventral motor/premotor (*t* = −3.02, *P* = .01, *d* = 0.87), and parietal (*t* = −2.27, *P* = .04, *d* = 0.65) cortices, Fig. 2c.

### Time-resolved decoding results

Neural distinctions between SVs and nSVs categories in the right fronto-temporal ROI (“MEG1322 + 1323,” “MEG1412 + 1413,” and “MEG1442 + 1443”) emerged 180 ms after target verb onset and remained significant up to 230 ms (AUC: *M* = 0.58, *SD* = 0.008), reaching a maximal decoding peak at 210 ms (AUC = 0.59), see Fig. 2d.

### RSA results

We evaluated the predictive performance of different conceptual models in explaining the structure of time-varying neural RDMs within the right fronto-temporal ROI. Each candidate model made explicit predictions regarding the expected dissimilarities between verb items in terms of (i) socialness, (ii) perceptual content, (iii) motor content, (iv) emotional valence, (v) arousal, (vi) concreteness, (vii) frequency, and (viii) AoA. Overall, information about word socialness predicted neural representations between 180–250 ms and 360–500 ms (all *P*_corr_ < .04, all *W*s > 86), outperforming all other models which failed to explain dissimilarities in time-resolved neural RDMs. See Fig. 2e.

## Discussion

We examined neural dynamics of SV processing, a conceptual domain-coding interpersonal behaviors. Relative to nSVs, SVs elicited stronger beta (20 Hz) power decreases in right fronto-temporal sensors at early stages. Key sources of this early beta effect involved the right superior ATL as well as parietal, pre/motor, and prefrontal cortices. Time‐resolved decoding of MEG sensor‐level data showed that SVs were discriminated from nSVs as early as 180 ms after word onset. RSA further revealed that these spatio-temporal patterns were uniquely explained by the words’ socialness, and were not accounted for by other semantic dimensions such as sensorimotor content, emotional valence, arousal, and concreteness, nor by lexical aspects such as verb frequency or AoA. These findings inform neurocognitive models of semantic cognition, as discussed further.

Time-frequency analysis revealed stronger beta power suppression for SVs compared to nSVs—a pattern that proved consistent across individuals. While no study has explored oscillatory modulations subserving social words, similar beta modulations have been observed during processing of action verbs (Moreno et al. 2013, Niccolai et al. 2016), emotional body language (Botta et al. 2024), action recognition (Pavlidou et al. 2014), and social interactions (Kourtis et al. 2010, Menoret et al. 2014), suggesting that SV processing recruits sociocognitive mechanisms shared across verbal and nonverbal domains. Using time-resolved decoding, we were able to identify social‐related information at early latencies in MEG sensor‐level data. Specifically, SVs could be decoded between 180 and 230 ms in right fronto-temporal sensors. Such early effect is consistent with the timing of lexico-semantic access during word reading (Carreiras et al. 2014, Dirani and Pylkkänen 2020) and the detection of social relevance (Proverbio et al. 2008, Schacht and Vrticka 2018, Lee Masson and Isik 2023, Moreau et al. 2023)—namely, biologically salient signals that naturally draw our attention to conspecifics. This indicates that lexical and interpersonal mechanisms are rapidly integrated during social-word retrieval. Thus, our findings suggest that social word recognition unfolds as fast as other social cues, including communicative gestures and emotions (Conty et al. 2012, Dima et al. 2018).

The beta effect underlying SVs was traced to the superior ATL as well as parietal, pre/motor, and prefrontal cortices, all in the right hemisphere. Neuroimaging studies consistently point to the right superior ATL as a central hub for processing social conceptual knowledge (Zahn et al. 2007, Pobric et al. 2016, Ralph et al. 2017). Indeed, the right ATL exhibits intracortical beta modulations during lexico-semantic retrieval (Abel et al. 2016, Cao et al. 2022), including words evoking social content (e.g. names of famous people). Atrophy and hypo-connectivity patterns in this region have been linked to impairments in mentalizing, person-specific knowledge, socio-emotional concept retrieval (Younes et al. 2022), and, more particularly, with deficits in social text comprehension (Liu et al. 2004, Birba et al. 2021). In addition to the ATL involvement, our findings align with fMRI studies on SV processing (Spunt et al. 2010, Lin et al. 2015), which implicate pre/motor, parietal, and prefrontal regions supporting action observation and mentalizing networks. Similarly, TMS evidence indicates that the motor system exhibits early enhanced activity during the semantic processing of socially exclusion adjectives (e.g. rejected, abandoned), supporting the reactivation of embodied social mechanisms (Vitale et al. 2023). Given the involvement of these areas in broader social cognition networks (Van Overwalle and Baetens 2009, Vogeley 2017), our results reinforce the notion that SV processing involves the recycling or neural reuse (Anderson 2010) of more general interpersonal mechanisms.

RSA further showed that these early fronto-temporal patterns were uniquely predicted by the socialness of words, as they were not explained by other semantic models based on sensorimotor properties, arousal, emotional valence, and concreteness, nor by lexical models based on frequency and AoA ratings. Previous studies have shown that social words constitute a distinct type of conceptual knowledge, forming a separate cluster within semantic space (Huth et al. 2016, Villani et al. 2019, Vargas and Just 2020). Moreover, fMRI studies have revealed selective activations for social compared to nonsocial matched words in the right superior ATL (Zahn et al. 2007, Olson et al. 2013, Binney et al. 2016), alongside correlations between such modulations and the concepts’ degree of social richness (Zahn et al. 2007). Our results extend these findings by showing that SV processing engages distinct oscillatory signatures in the beta band. The topography of beta power decreases varies depending on stimulus type (e.g. words versus faces), suggesting material-specific memory reactivation (Fellner et al. 2019). According to the “information via desynchronization” hypothesis (Hanslmayr et al. 2012, 2016), beta desynchronization facilitates selective memory retrieval by increasing information richness. This mechanism allows cortical assemblies to encode stimulus-specific information through more complex and varied neural firing patterns. We propose that the observed beta desynchronization for SVs reflects a similar material-specific reactivation process, supporting the retrieval of social conceptual knowledge by enhancing the richness of social memory traces in right fronto-temporal areas.

Lastly, no effects were observed in the theta band. Theta oscillations mediate developmental changes in networks engaged by social stimuli (Jones et al. 2015, van der Velde et al. 2021), although such evidence comes mainly from videos rather than linguistic material. Thus, we surmise that theta effects might be distinctly sensitive to the visualization of interpersonal events, pointing to the potential role of beta oscillations in cross-modal social processing. This proposed dissociation could inspire comparisons between presentation modalities in future socio-cognitive MEG research.

Altogether, our results inform neural models of social conceptual knowledge (Ralph et al. 2017, Binney and Ramsey 2020) and of social cognition in general (Adolphs 2010, Frith and Frith 2010). While previous works—primarily based on fMRI findings—have illuminated the brain areas mediating socialness effects, they overlook the spectral and temporal dynamics enabling timely networking and communication in specific frequency bands (Fries 2015). Based on present findings, we propose that beta dynamics underpin early stages of SV processing—an effect that may conceivably extend to other lexical categories (e.g. social nouns or adjectives). As in previous text-level research (Birba et al. 2021, Lopes da Cunha et al. 2023), social words in our study consistently engaged brain systems known to subserve more general socio-cognitive phenomena, such as social salience detection, mentalizing, and action recognition. This strengthens the notion that retrieving social words, including SVs, recycles interpersonal mechanisms that facilitate the processing of socially relevant stimuli.

The present study is not without limitations. First, our sample size was small. Although previous M/EEG studies using social stimuli have yielded replicable results with similar or even smaller sample sizes (Proverbio et al. 2008, Kourtis et al. 2010, Pavlidou et al. 2014), it would be desirable to test our approach with more participants. Second, the absence of socio-cognitive measures limits our understanding of the observed effects. Future studies on SVs should incorporate participants’ individual ratings on relevant domains [e.g. empathy, metalizing, and social skills—e.g. via standardized measures (Davis 1980)], to explore their association with beta patterns. Furthermore, our study focused exclusively on verbs. To obtain a more comprehensive understanding of the neural underpinnings of social language processing, future research could include social nouns or adjectives. Lastly, while the use of identical sentence structures for all stimuli aided in controlling potential morphosyntactic confounds, it would be informative to explore socialness effects using more naturalistic experimental designs, better reflecting real-world language processing (Lopes da Cunha et al. 2023).

## Conclusions

Processing of SVs, a key linguistic category for communicating social concepts, appears to involve distinct spectro-temporal, and spatial signatures in the human brain. In particular, early beta oscillations, traceable to the right superior ATL and other key social brain regions, seem differentially modulated during access to these words. These insights not only extend current theoretical accounts of semantic memory and social cognition, but also pave the way for the potential identification of oscillatory language markers in patients with fronto-temporal damage.
